# External validity of phase III trials on vaccines against SARS-CoV-2 to a middle-aged and elderly Western European population

**DOI:** 10.1007/s10654-021-00729-5

**Published:** 2021-02-26

**Authors:** Natalie Terzikhan, Albert Hofman, Jaap Goudsmit, Mohammad Arfan Ikram

**Affiliations:** 1 Department of Epidemiology, Erasmus University Medical Center, Wytemaweg 80, 3015 CN Rotterdam, the Netherlands; 2 Department of Epidemiology, Harvard T.H. Chan School of Public Health, Boston, MA USA; 3 Human Immunomics Initiative, Department of Epidemiology, Harvard T.H. Chan School of Public Health and Human Vaccines Project, Boston, MA USA; 4 Department of Immunology and Infectious Diseases, Harvard T.H. Chan School of Public Health, Boston, MA USA

**Keywords:** SARS-CoV-2, Vaccines, Phase III trials, Generalizability, External validity, Epidemiology

## Abstract

Initial results from various phase-III trials on vaccines against SARS-CoV-2 are promising. For proper translation of these results to clinical guidelines, it is essential to determine how well the general population is reflected in the study populations of these trials. This study was conducted among 7162 participants (age-range: 51–106 years; 58% women) from the Rotterdam Study. We quantified the proportion of participants that would be eligible for the nine ongoing phase-III trials. We further quantified the eligibility among participants at high risk to develop severe COVID-19. Since many trials were not explicit in their exclusion criterion with respect to ‘acute’ or ‘unstable preexisting’ diseases, we performed two analyses. First, we included all participants irrespective of this criterion. Second, we excluded persons with acute or ‘unstable preexisting’ diseases. 97% of 7162 participants was eligible for any trial with eligibility for separate trials ranging between 11–97%. For high-risk individuals the corresponding numbers were 96% for any trial with separate trials ranging from 5–96%. Importantly, considering persons ineligible due to ‘acute’ or ‘unstable pre-existing’ disease drastically dropped the eligibilities for all trials below 43% for the total population and below 36% for high-risk individuals. The eligibility for ongoing vaccine trials against SARS-CoV-2 can reduce by half depending on interpretation and application of a single unspecified exclusion criterion. This exclusion criterion in our study would especially affect the elderly and those with pre-existing morbidities. These findings thus indicate the difficulty as well as importance of developing clinical recommendations for vaccination and applying these to the appropriate target populations. This becomes especially paramount considering the fact that many countries worldwide have initiated their vaccination programs by first targeting the elderly and most vulnerable persons.

**Supplementary Information:**

The online version contains supplementary material available at 10.1007/s10654-021-00729-5.

## Introduction

Several phase III trials on vaccines against SARS-CoV-2 are ongoing and initial results highly promising. A major issue of these phase III trials is to what extent the included study population is representative of the intended (or target) population, i.e. external validity.

For these trials, the intended target population is initially comprised of high-risk individuals usually considered to be elderly persons as well as those with comorbidities, and ultimately the entire world population. It remains unclear whether these target populations are representatively recruited into ongoing trials. This information is pivotal, since clinical recommendations for any approved vaccine should incorporate the proper target populations for which these vaccines have shown efficacy, and also determine those populations not sufficiently represented in the trials.

We sought to quantify the external validity of the various ongoing trials to a middle-aged and elderly West-European population from the Rotterdam Study. Specifically, we were interested to quantify what proportion of this study population would be eligible to participate in these trials and how many of those eligible are from high-risk categories.

## Methods

Extensive methods are available in the Online Resource.

Briefly, for this study we screened www.clinicaltrials.gov for ongoing phase III trials focused on vaccine development against SARS-CoV-2 and COVID-19.

The group at high risk of severe COVID-19 was defined according to the criteria of the Dutch National Institute for Public Health and the Environment (RIVM) [1], and included participants aged 70 or higher, and participants with asthma and COPD, diabetes, cancer, participants with current use of antineoplastic and immunosuppressive agents, obesity, end-stage kidney disease, liver steatosis and cirrhosis and cardiac diseases.

We carried out our analyses in the population-based Rotterdam Study [2] and used data collected from 2009 to 2014, which yielded 7162 persons (mean age 70 years (SD 9.8), 58% women) for analysis (Online Resource Table 1). These calendar-years were chosen such to maximize the number of living participants as well as their available data. Data on the comorbidities was ascertained during the in-person examinations complemented by automated linkage of medical and pharmacy records to our study database.

We applied eligibility criteria from each separate trial to our study population and calculated the following proportions: the proportion of participants eligible for any trial, and for each trial separately, the number of high-risk individuals in our study eligible for any trial, and for each trial separately.

We performed two complementary analyses and calculated the abovementioned proportions in each analysis separately. These two analyses differed with respect to the interpretation of an eligibility criterion that was not always explicitly specified in the various trial protocols. This criterion was often stated as follows: ‘*preexisting (un)stable disease*’, ‘*an acute course of disease*’, or ‘*other medical or psychiatric condition or laboratory abnormality that may increase the risk of study participation or, in the investigator’s judgment, make the participant inappropriate for the study*’. In our dataset, we operationalized this criterion as follows: diagnosis of dementia, diagnosis of moderate to severe COPD, current clinically significant depressive symptoms, abnormal kidney function, current liver disease (defined as liver steatosis, and liver cirrhosis) or a new diagnosis within the preceding three months for the following conditions: stroke, cancer, (including antineoplastic agents), diabetes mellitus, COPD, cardiac disease (heart failure, myocardial infarct, atrial fibrillation, and revascularisation). In the first analysis, we included everyone as eligible, who met this operationalization and in the second analysis, we excluded anyone who met this operationalization. The general characteristics of individuals with or without acute or unstable disease are presented in Online Resource Tables 2 and 3).

Finally, in sensitivity analyses we incrementally restricted the study population to persons aged over 60, 70, and 80 years (Online Resource Fig. 1).

**Fig. 1:**
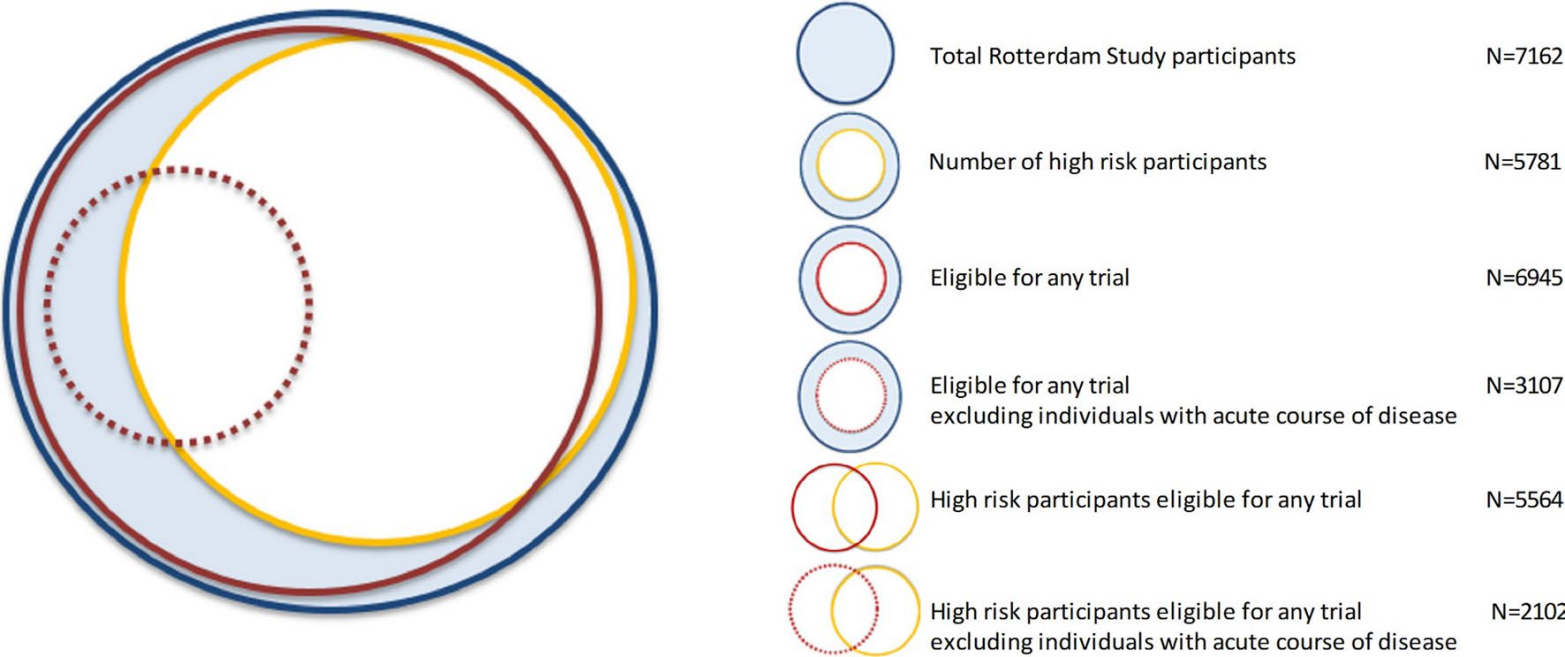
Venn diagram for the proportion of eligible Rotterdam Study participants for any trial. The colours are coded as follows: blue circle: total study population; yellow circle: persons at high risk of severe COVID-19; red circle: persons eligible for any trial in the first analysis; red dotted circle: persons eligible for any trial in the second analysis, in which individuals with acute course of disease were excluded

## Results

Table 1 presents the exclusion criteria from the 9 included trials, and we note that seven of these mentioned the eligibility criterion of ‘acute’ or ‘unstable preexisting’ disease without further specification. Therefore, we performed two analyses to calculate the proportion of the eligible participants for each trial. In the first analysis, we included all eligible participants, including those who met our operationalization of the eligibility criterion of ‘acute’ or ‘unstable preexisting’ in the seven trials that mentioned this criterion without further specification. In the second analysis, we repeated the first analysis but now excluding those who met our operationalization of ‘acute’ or ‘unstable preexisting’ criterion.

**Table 1:** Overview of the included trials with the selection of exclusion criteria per trial, for which data from the Rotterdam Study was available

Trial sponsor (collaborator) Clinical Trials.gov identifier		
**ModernaTX, Inc** (Biomedical advanced research and development authority and NIAID)	**Janssen vaccines and prevention B.V**	**Butantan Institute** (Sinovac Life Sciences Co., Ltd.)
NCT04470427	NCT04505722	NCT04456595
Immunodeficient state and therapy*	Immunodeficient state and therapy*	Immunodeficient state and therapy*
Acute or unstable disease, not further specified	Acute or unstable disease, not further specified	Current diagnosis/treatment for cancer
		Alcohol dependency
		Depressive symptoms
		Dementia diagnosis
		Acute or unstable disease, not further specified
**AstraZeneca** (Iqvia Pty Ltd)	**BioNTech SE** (Pfizer)	**Novavax**
NCT04516746	NCT04368728	NCT04583995
Immunodeficient state and therapy*	Immunodeficient state and therapy*	Immunodeficient state and therapy*
History of malignancy^b^	Age > 85 years	Current diagnosis/treatment for cancer
Acute or unstable disease, not further specified	Dementia diagnosis	Alcohol dependency
	Depression	Continuous use of anticoagulants^d^
	Acute or unstable disease, not further specified	History of chronic neurological disorders that have required prior specialist medical review^a^
		Age > 84 years
		Acute or unstable disease, not further specified
**Gamaleya Research Institute & Health Ministry of the Russian Federation** (Government of the city of Moscow and CRO)	**China National Biotec Group Company Limited** (G42 Healthcare company, Abu Dhabi Health Services Company, Wuhan Institute of Biological Products Co., Ltd and Beijing Institute of Biological Products Co., Ltd)	**NPO Petrovax** (CanSino Biologics Inc.)
NCT04530396	NCT04510207	NCT04540419
Immunodeficient state and therapy*	Immunodeficient state and therapy*	Immunodeficient state and therapy*
History of neoplasms	DiaBP > 90 mmHg	History of malignancies^c^
Alcohol dependency	SysBP > 150 mmHg	Age > 85 years
Acute stroke the previous year	Dementia diagnosis	History of diabetes mellitus
Acute cardiac disease in the previous year	Acute or unstable disease, as specified in the footnote^e^	18.5 < BMI > 30
Acute or unstable disease, not further specified		SysBP > 139 mmHg DiaBP > 90 mmHg
		SysBP < 100 mmHg DiaBP < 60 mmHg
		Acute or unstable disease, as specified in the footnote^e^

*BMI* body mass index, *DiaBP* diastolic blood pressure, *SysBP* systolic blood pressure

*Immunosuppressive and immunomodulatory medications: Immunosuppressive medications, corticosteroid use and antineoplastic agents; ICD10-codes: L04, L01, H01

^a^History of chronic neurological disorders that have required prior specialist medical review: Dementia, Parkinson Disease, Stroke in the previous year

^b^Except childhood cancers and prostate cancer and uterine cervical carcinoma

^c^Including history of lymphoma and history of haematopoietic cancer

^d^ICD10- codes: B01AA and B01AE

^e^Acute or unstable disease is defined as: Dementia diagnosis, diagnosis of moderate to severe Chronic obstructive pulmonary disease (COPD), current depressive symptoms, abnormal kidney function (defined as estimated Glomerular Filtration Rate < 60 millilitre/minute), current liver disease (defined as liver steatosis, and liver cirrhosis). Diagnosis of the following within the previous 3 months: stroke, cancer (including antineoplastic agents), diabetes mellitus, COPD, cardiac disease (heart failure, myocardial infarct, atrial fibrillation, and revascularisation)

97% (N = 6945) of the total Rotterdam Study participants would be eligible for any trial in the first analysis, while this percentage dropped to 43% (N = 3107) in the second analysis. Among the 5781 participants at high-risk of severe COVID-19, 96% (N = 5564) would be eligible for any trial in the first analysis and 36% (N = 2102) in the second analysis (Fig. 1).

**Fig. 2:**
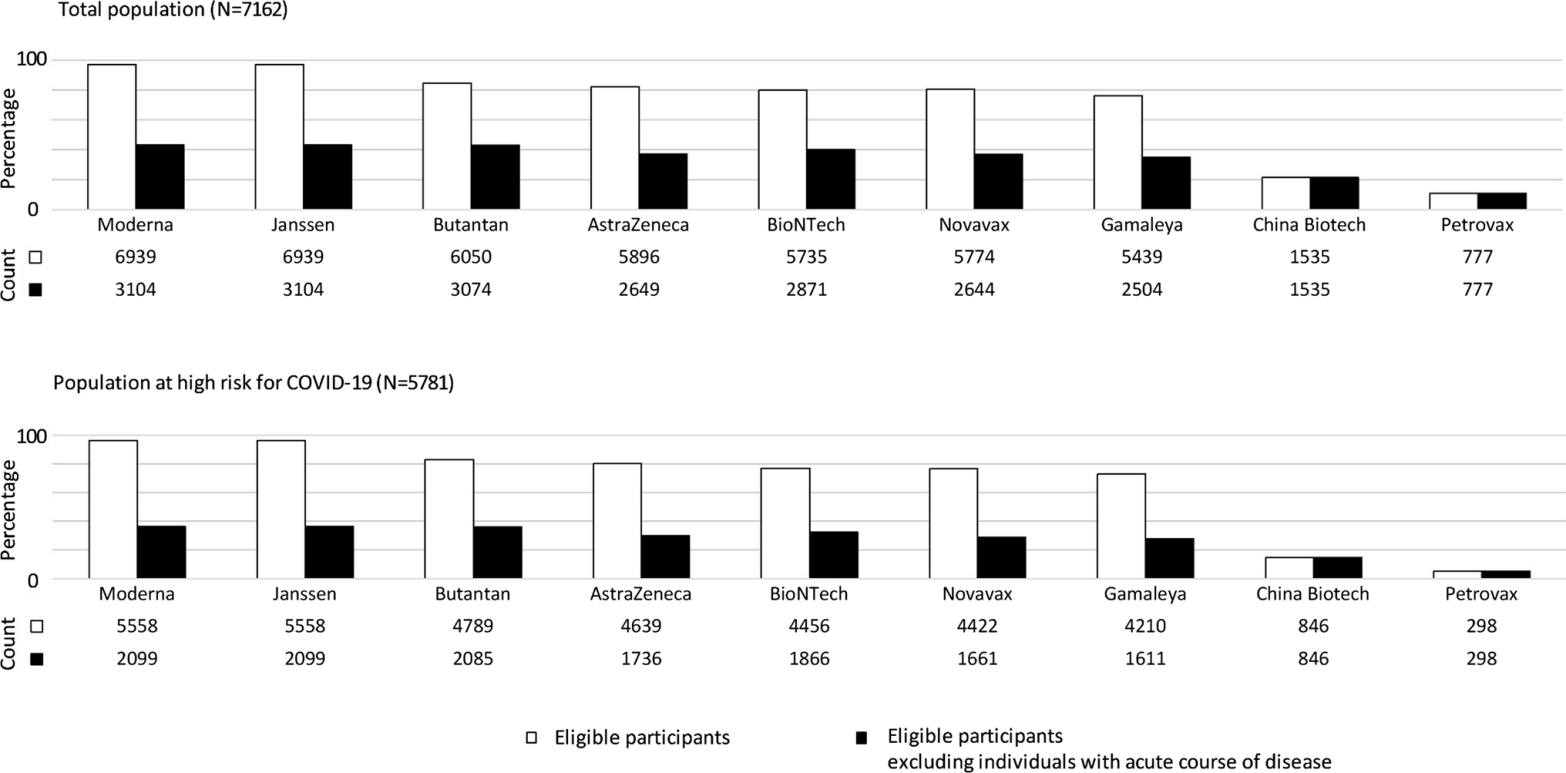
The number and proportion of eligibility from the Rotterdam Study population for ongoing clinical phase III trials on a vaccine against SARS-CoV-2. White bars indicate data from the first analysis; black bars indicate data from the second analysis. The difference between these two analyses is the operationalization, and thus inclusion or exclusion, of the eligibility criteria ‘(un)stable disease’, ‘acute course of disease’, or ‘other condition increasing risk of participation’. **a** total population of the Rotterdam Study and **b** individuals at high risk of severe COVID-19

Figure 2a shows the percentages for the two analyses for each trial separately. Whereas in the first analysis the proportion included for the most inclusive trial was 97%, this number dropped considerably in the second analysis to 43%. Figure 2b shows the corresponding numbers from participants at high-risk of severe COVID-19. Finally, sensitivity analyses revealed similar patterns at various age cut-offs (Online Resource Fig. 1).

## Discussion

In a middle-aged and elderly population in the Netherlands from predominantly West-European descent, we found that 97% of this population would be eligible to participate in any of the nine currently ongoing vaccine trials against SARS-CoV-2. For persons at high-risk of severe COVID-19, the eligibility for any trial was 96%. Importantly, applying stricter exclusion based on the criterion ‘acute’ or ‘unstable preexisting’ disease drastically reduced the eligibility for any trial to 43% of the entire study population and 36% of the high-risk individuals.

Clinical trials are often considered the golden standard in efficacy research, due to several strengths by design with respect to internal validity. In contrast, whether findings from clinical trials are adequately translated to clinical practice also depends on their external validity. External validity is the extent to which findings from one study are applicable to target populations not represented in the actual study population. To properly gauge the actual study population, it is crucial that clinical trials explicitly report the setting of the trial, the exact intervention, the inclusion and exclusion criteria, and the characteristics of the actually recruited population [3, 4] in [5]. For the ongoing clinical trials, any effective vaccine can be considered effective in similar settings as the original trial. The judgement whether that vaccine will be also effective in other settings, i.e. external validity, is based on prior knowledge, biological plausibility, statistical considerations and eligibility criteria of the original trial [3]. Many countries worldwide have prioritized the elderly and those most vulnerable in their vaccination campaigns, indicating that policy-makers consider the external validity of the ongoing trials to these populations sufficient.

The population of the Rotterdam Study is a lower-middle class population of primarily European descent. Previously, this population has shown good generalizability to the population of the Netherlands [6–8]. Another important metric in this regard is the response rate, which has continuously exceeded 70% for the Rotterdam Study [2]. This is a major strength of our study which makes it not only population-based but also population-representative, and far exceeds response rates for other larger efforts ongoing worldwide [9]. Notwithstanding these considerations, the drastic drop in eligibility for the stricter criterion in the second analysis would likely have been of the same magnitude in any other population, irrespective of geographical or ethnic setting or response rate.

In conclusion, we found that eligibility for ongoing vaccine trials against SARS-CoV-2 can reduce by half depending on interpretation and application of a single unspecified exclusion criterion. This exclusion criterion in our study would especially affect the elderly and those with pre-existing morbidities. These findings thus indicate the difficulty as well as importance of developing clinical recommendations for vaccination and applying these to the appropriate target populations. This becomes especially paramount considering the fact that many countries worldwide have initiated their vaccination programs by first targeting the elderly and most vulnerable persons.

## Supplementary Information

Below is the link to the electronic supplementary material.

Supplementary file1 (DOCX 356 KB)

## Data Availability

Data can be obtained upon request. Requests should be directed towards the management team of the Rotterdam Study (secretariat.epi@erasmusmc.nl), which has a protocol for approving data requests. Because of restrictions based on privacy regulations and informed consent of the participants, data cannot be made freely available in a public repository.
