# Supplementary material for: External validity of phase III trials on vaccines against SARS-CoV-2 to a middle-aged and elderly Western European population
Source: Eur J Epidemiol. 2021 Feb 26;36(3):319–24. doi: 10.1007/s10654-021-00729-5 (PMC7906827; doi:10.1007/s10654-021-00729-5)
Supplement: Supplementary file 1 — Supplementary file1 (DOCX 356 KB) [file 10654_2021_729_MOESM1_ESM.docx]

**Online Resource**

**European Journal of Epidemiology**

**External validity of phase III trials on vaccines against SARS-CoV-2 to a middle-aged and elderly Western European population**

Natalie Terzikhan PhD^1^, Albert Hofman PhD^2^, Jaap Goudsmit PhD^3,4^, M. Arfan Ikram PhD^1^

1. Department of Epidemiology, Erasmus University Medical Center, Wytemaweg 80, 3015 CN, Rotterdam, the Netherlands

2. Department of Epidemiology, Harvard T.H. Chan School of Public Health, Boston, MA, USA

3. Human Immunomics Initiative, Department of Epidemiology of the Harvard T.H. Chan School of Public Health & Human Vaccines Project, Boston, MA, USA

4. Department of Immunology and Infectious Diseases, Harvard T.H. Chan School of Public Health, Boston, MA, USA

**Address for correspondence:** M.A. Ikram, Department of Epidemiology, Erasmus University Medical Center, Wytemaweg 80, 3015 CN, Rotterdam, the Netherlands. Telephone: +31107043939. Email: [m.a.ikram@erasmusmc.nl](mailto:m.a.ikram@erasmusmc.nl)

**Content:**

**Methods**

**Results**

**Online Resource Table 1** General characteristics of the study population.

**Online Resource Table 2** General characteristics of the study population, stratified by health status.

**Online Resource Table 3** General characteristics of the study population at high risk for severe COVID-19, stratified by health status.

**Online Resource Figure 1** The number and proportion of eligibility from the Rotterdam Study population for ongoing clinical phase III trials on a vaccine against SARS-CoV-2.

**References**

**Methods**

*Selection of trials and high-risk groups*

For this study, we screened the website [www.clinicaltrials.gov](http://www.clinicaltrials.gov) for ongoing phase III preventive trials focused on vaccine development against SARS-CoV-2 and COVID-19. The following key terms were used for the search: Phase III, sponsor, recruiting, efficacy, SARS-CoV-2, and COVID-19. If studies from the same sponsor or the same intervention had different registrations, we only included one study. Clinical trials that used a drug or any other existing vaccine in the intervention group e.g. the Bacillus Calmette-Guérin (BCG) vaccine, were excluded.

The group at high risk of severe COVID-19 was defined according to the criteria of the Dutch National Institute for Public Health and the Environment (RIVM) [1], which largely draws from information from the WHO. This group included participants aged 70 or higher, and participants with asthma and COPD, diabetes, cancer, participants with current use of antineoplastic and immunosuppressive agents, obesity (BMI >30), end-stage kidney disease with estimated Glomerular Filtration Rate (eGFR) <15 milliliter/minute, liver steatosis and cirrhosis, cardiac diseases (including myocardial infarction, heart failure, atrial fibrillation and revascularization).

*Study population*

The present study was embedded within the Rotterdam Study, an ongoing prospective population-based cohort study that investigates the occurrence of chronic diseases and risk factors in middle-aged and elderly participants. The objective and methods of this cohort have been published previously [2]. In short, the Rotterdam Study (RS) includes 14,926 participants aged ≥ 45 years, living in Ommoord, a well-defined suburb of the city of Rotterdam, the Netherlands, and encompasses three recruitment waves, indicated as RS I, RS II, RS III. Study entry for these waves was in 1990, 2000, and 2005, respectively. After study entry, follow-up examinations take place every 4 to 5 years. At study entry and each follow-up examination, all participants are initially interviewed at home followed by an extensive set of examinations performed at a specially built research facility in the study district. Trained research assistants collect follow-up information from medical records of nursing homes, general practitioners and hospitals. The study was approved by the medical ethics committee of Erasmus Medical Center, Rotterdam. All participants included in this study provided their written consent for study participation and access to their medical files. For this study we used the data collected from 2009 to 2014, which corresponds to the 5^th^ wave of the first cohort (RSI-5), the 3^rd^ wave of the second cohort (RSII-3) and the second wave of the third cohort (RSIII-2). These examinations were chosen such to maximise the number of participants for this analysis as well as their available data.

*Ascertainment of clinical data and comorbidities*

Data on the comorbidities was ascertained during the in-person examinations complemented by automated linkage of general practitioners’ (GP) and pharmacy records to our study database. The health care system in the Netherlands is organized such that the GP by law acts as gate-keeper for all contacts of patients with care-givers. In return, all care-givers provide relevant copies and letters to GPs of any care provided to the patient. The medical records of the GP are therefore both complete and accurate regarding formal care provided to patients.

Asthma and COPD cases were defined as persons having a physician’s diagnosis reported in their medical charts. Case identification and validation of asthma and COPD was described previously [3, 4]. Cancer cases were defined based on GP records and through linkage our study database with the national registry of histo- and cytopathology in the Netherlands. All cancers were classified via ICD-10 and ICPC-2 and were confirmed by pathology. Cardiac outcomes included coronary heart disease, heart failure and cardiac arrhythmia. Case identification and validation of the cardiac outcomes was described extensively elsewhere [5]. Prevalent depressive symptoms were assessed by interview using the validated Center for Epidemiologic Studies Depression Scale (CES-D). With scores ranging between 0 (no depressive symptoms) and 60 (many symptoms) [6]. A cut-off of 16 or higher is used as a screening tool for detecting major depression. The diagnosis of dementia followed a three-step protocol, first all participants underwent a cognitive test. Next screen-positive participants underwent additional cognitive testing. Finally, participants with results suggestive of dementia underwent detailed examination and had their medical charts reviewed to confirm diagnosis and type of dementia [7]. Diabetes mellitus type 2 was defined as having a fasting blood glucose ≥7.0 mmol/l, a non-fasting glucose ≥11.1 mmol/l or use of blood glucose lowering medication [8]. Liver cirrhosis was detected by the liver stiffness measures (LSM). LSM were performed using a fibroScan. Clinically relevant cirrhosis was defined as LSM P13.0 kPa. For liver steatosis, abdominal ultrasound exam was performed by a certified and experienced technician. Images were stored digitally and re-evaluated by an experienced hepatologist. Diagnosis of steatosis was defined as the presence or absence of a hyper-echogenec liver parenchyma [9]. Abnormal kidney function was defined as an eGFR ≤ 60 milliliter/minute. Obesity was defined as a BMI ≥ 30 Kg/M^2^. The diagnosis of Parkinson’s Diseases followed a two-step protocol, this was described extensively elsewhere [10]. Smoking status was assessed during home interviews. Cases with stroke were identified based on self-report, medical chart and hospitalization data [11]. Current medication use was gathered through automated linkage with the pharmacies in the study district. Alcohol dependency was assessed during the home interviews using the Cut down, Annoyed, Guilty and Eye opener (CAGE) questionnaire, which is a validated scale to measure mostly psychological alcohol dependency [12]. The score ranges between 0-4 with cut-off of 3 used as positive screen for alcohol dependency [13].

*Data analysis*

Data analysis was performed in SPSS and R statistical software. After summarizing the exclusion criteria for each trial (using the information from the study protocol and the website [www.clinicaltrials.gov](http://www.clinicaltrials.gov)), we applied those criteria to our study population and quantified the number of persons eligible for those trials. We then calculated the following proportions: the proportion of participants of the Rotterdam Study eligible for any trial, the proportion of participants eligible for each trial separately, the number of high-risk individuals within the Rotterdam Study eligible for any trial, and the number of high-risk individuals eligible for each trial separately. If data on any variable was missing we considered that person to be eligible for the trial with respect to that variable.

We performed two complementary analyses and calculated the abovementioned proportions in each analysis separately. These two analyses differed with respect to the interpretation of an eligibility criterion that was not always explicitly specified in the various trial protocols. This criterion was often stated as follows: ‘*preexisting (un)stable disease*’, ‘*an acute course of disease*’, or ‘*other medical or psychiatric condition or laboratory abnormality that may increase the risk of study participation or, in the investigator’s judgment, make the participant inappropriate for the study*’. In our dataset, we operationalized this criterion as follows: diagnosis of dementia, diagnosis of moderate to severe COPD, current clinically significant depressive symptoms, abnormal kidney function, current liver disease (defined as liver steatosis, and liver cirrhosis) or a new diagnosis within the preceding three months for the following conditions: stroke, cancer, (including antineoplastic agents), diabetes mellitus, COPD, cardiac disease (heart failure, myocardial infarct, atrial fibrillation, and revascularisation). In the first analysis, we included everyone as eligible, who met this operationalization and in the second analysis, we excluded anyone who met this operationalization.

Finally, in sensitivity analyses we incrementally restricted the study population to persons aged over 60, 70, and 80 years.

**Results**

**Online Resource Table 1 General characteristics of the study population.**

|  | **Overall** | **Normal risk for severe COVID-19** | **High risk for severe COVID-19** |
| --- | --- | --- | --- |
| Number | 7162 | 1381 | 5781 |
| Age at interview, years | 70.4 (9.8) | 61.9 (5.0) | 72.4 (9.5) |
| Age over 60 years | 5945 (83.0) | 852 (61.7) | 5093 (88.1) |
| Age over 70 years | 3531 (49.3) | 0 (0.0) | 3531 (61.1) |
| Age over 80 years | 1285 (17.9) | 0 (0.0) | 1285 (22.2) |
| Women | 4170 (58.2) | 861 (62.3) | 3309 (57.2) |
| Genetic ancestry, European* | 6100 (97.5) | 1173 (96.5) | 4927 (97.7) |
| Systolic blood pressure, mmHg | 264.8 (298.6) | 210.1 (247.5) | 277.8 (308.1) |
| Diastolic blood pressure, mmHg | 212.7 (319.0) | 162.6 (261.8) | 224.7 (330.1) |
| Hypertension | 5130 (75.6) | 627 (48.1) | 4503 (82.1) |
| Smokers | 906 (12.7) | 198 (14.3) | 708 (12.2) |
| Current medication use |  |  |  |
| Immunosuppressants | 66 (9.6) | 0 (0.0) | 66 (9.7) |
| Antithrombotic agents | 464 (67.5) | 7 (100.0) | 457 (67.2) |
| Corticosteroids | 143 (20.8) | 0 (0.0) | 143 (21.0) |
| Antineoplastic agents | 14 (2.0) | 0 (0.0) | 14 (2.1) |
| Alcohol dependency | 79 (1.1) | 18 (1.3) | 61 (1.1) |
| Prevalent diseases |  |  |  |
| Diabetes | 655 (9.4) | 0 (0.0) | 655 (11.7) |
| Diabetes within 3 prior months | 426 (6.8) | 159 (11.6) | 267 (5.4) |
| Dementia | 99 (1.4) | 0 (0.0) | 99 (1.7) |
| Depressive symptoms | 759 (10.7) | 102 (7.4) | 657 (11.5) |
| Abnormal kidney function | 907 (15.0) | 54 (4.4) | 853 (17.7) |
| Liver cirrhosis or steatosis | 2146 (42.7) | 0 (0.0) | 2146 (54.6) |
| Cancer | 1211 (73.8) | 131 (78.4) | 1080 (73.2) |
| Cancer within 3 prior months | 14 (0.9) | 0 (0.0) | 14 (0.9) |
| Stroke within 12 prior months | 41 (0.6) | 4 (0.3) | 37 (0.7) |
| Stroke within 3 prior months | 6 (0.1) | 1 (0.1) | 5 (0.1) |
| Parkinson’s disease | 25 (0.4) | 1 (0.1) | 24 (0.4) |
| Cardiac disease within 3 prior months | 345 (4.8) | 99 (7.2) | 246 (4.3) |
| Moderate to severe COPD | 419 (5.9) | 0 (0.0) | 419 (7.2) |
| COPD within 3 prior months | 22 (1.5) | 0 (0.0) | 22 (1.6) |
| Values are absolute numbers (%) or means (standard deviation). The high-risk group was based on recommendations from the RIVM and WHO, and included participants aged over 70 years, and participants with asthma and COPD, diabetes, cancer, participants with current use of antineoplastic and immunosuppressive agents, obesity (body mass index >30 kg/m^2^), end-stage kidney disease with estimated glomerular filtration rate <15 milliliters/minute, liver steatosis or cirrhosis, cardiac diseases (including myocardial infarction, heart failure, atrial fibrillation and revascularization).  COPD: Chronic obstructive pulmonary disease.  *Genetic ancestry data was available in a random set of 87% of this study population. | | | |

**Online Resource Table 2 General characteristics of the study population, stratified by health status.**

|  | **Overall** | **Healthy** | **Acute or unstable disease** |
| --- | --- | --- | --- |
| Number | 7162 | 3204 | 3958 |
| Age at interview, years | 70.4 (9.8) | 70.13 (9.8) | 70.58 (9.8) |
| Age over 60 years | 5945 (83.0) | 2631 (82.1) | 3314 (83.7) |
| Age over 70 years | 3531 (49.3) | 1555 (48.5) | 1976 (49.9) |
| Age over 80 years | 1285 (17.9) | 544 (17.0) | 741 (18.7) |
| Women | 4170 (58.2) | 1907 (59.5) | 2263 (57.2)* |
| Genetic ancestry, European^ | 6100 (97.5) | 2698 (97.2) | 3402 (97.8) |
| Systolic blood pressure, mmHg | 264.8 (298.6) | 314.13 (344.2) | 224.79 (248.8)* |
| Diastolic blood pressure, mmHg | 212.7 (319.0) | 266.68 (367.1) | 169.03 (266.0)* |
| Hypertension | 5130 (75.6) | 2069 (69.8) | 3061 (80.0)* |
| Smokers | 906 (12.7) | 362 (11.3) | 544 (13.7)* |
| Current medication use |  |  |  |
| Immunosuppressants | 66 (9.6) | 34 (12.1) | 32 (7.9)* |
| Antithrombotic agents | 464 (67.5) | 181 (64.4) | 283 (69.7) |
| Corticosteroids | 143 (20.8) | 66 (23.5) | 77 (19.0) |
| Antineoplastic agents | 14 (2.0) | 0 (0.0) | 14 (3.4)* |
| Alcohol dependency | 79 (1.1) | 32 (1.0) | 47 (1.2) |
| Prevalent diseases |  |  |  |
| Diabetes | 655 (9.4) | 217 (7.0) | 438 (11.4)* |
| Diabetes within 3 prior months | 426 (6.8) | 0 (0.0) | 426 (12.5)* |
| Dementia | 99 (1.4) | 0 (0.0) | 99 (2.5)* |
| Depressive symptoms | 759 (10.7) | 0 (0.0) | 759 (19.4)* |
| Abnormal kidney function | 907 (15.0) | 0 (0.0) | 907 (25.6)* |
| Liver cirrhosis or steatosis | 2146 (42.7) | 0 (0.0) | 2146 (69.5)* |
| Cancer | 1211 (73.8) | 521 (72.7) | 690 (74.6) |
| Cancer within 3 prior months | 14 (0.9) | 0 (0.0) | 14 (1.5)* |
| Stroke within 12 prior months | 41 (0.6) | 10 (0.3) | 31 (0.8)* |
| Stroke within 3 prior months | 6 (0.1) | 0 (0.0) | 6 (0.2) |
| Parkinson’s disease | 25 (0.4) | 6 (0.2) | 19 (0.5) |
| Cardiac disease within 3 prior months | 345 (4.8) | 0 (0.0) | 345 (8.7)* |
| Moderate to severe COPD | 419 (5.9) | 0 (0.0) | 419 (10.6)* |
| COPD within 3 prior months | 22 (1.5) | 0 (0.0) | 22 (2.3)* |
| Values are absolute numbers (%) or means (standard deviation). Acute or unstable disease is defined as: Dementia diagnosis, diagnosis of moderate to severe Chronic obstructive pulmonary disease (COPD), current depressive symptoms, abnormal kidney function (defined as estimated Glomerular Filtration Rate <60 millilitre/minute), current liver disease (defined as liver steatosis, and liver cirrhosis). Diagnosis of the following within the previous 3 months: stroke, cancer (including antineoplastic agents), diabetes mellitus, COPD, cardiac disease (heart failure, myocardial infarct, atrial fibrillation, and revascularisation).  ^Genetic ancestry data was available in a random set of 87% of this study population. | | | |
| *P-value < 0.05 | | | |

**Online Resource Table 3 General characteristics of the study population at high risk for severe COVID-19, stratified by health status.**

|  | **Overall at high risk for severe COVID-19** | **Healthy** | **Acute or unstable disease** |
| --- | --- | --- | --- |
| Number | 5781 | 2199 | 3582 |
| Age at interview, years | 72.4 (9.5) | 73.81 (9.2) | 71.55 (9.6)* |
| Age over 60 years | 5093 (88.1) | 1997 (90.8) | 3096 (86.4)* |
| Age over 70 years | 3531 (61.1) | 1555 (70.7) | 1976 (55.2)* |
| Age over 80 years | 1285 (22.2) | 544 (24.7) | 741 (20.7)* |
| Women | 3309 (57.2) | 1288 (58.6) | 2021 (56.4) |
| Genetic ancestry, European^ | 4927 (97.7) | 1851 (97.4) | 3076 (98.0) |
| Systolic blood pressure, mmHg | 277.8 (308.1) | 365.99 (372.2) | 223.67 (245.4)* |
| Diastolic blood pressure, mmHg | 224.7 (330.1) | 318.71 (399.8) | 166.96 (262.6)* |
| Hypertension | 4503 (82.1) | 1624 (81.0) | 2879 (82.7) |
| Smokers | 708 (12.2) | 231 (10.5) | 477 (13.3)* |
| Current medication use |  |  |  |
| Immunosuppressants | 66 (9.7) | 34 (12.3) | 32 (7.9) |
| Antithrombotic agents | 457 (67.2) | 176 (63.8) | 281 (69.6) |
| Corticosteroids | 143 (21.0) | 66 (23.9) | 77 (19.1) |
| Antineoplastic agents | 14 (2.1) | 0 (0.0) | 14 (3.5)* |
| Alcohol dependency | 61 (1.1) | 16 (0.7) | 45 (1.3) |
| Prevalent diseases |  |  |  |
| Diabetes | 655 (11.7) | 217 (10.3) | 438 (12.6)* |
| Diabetes within 3 prior months | 267 (5.4) | 0 (0.0) | 267 (8.8)* |
| Dementia | 99 (1.7) | 0 (0.0) | 99 (2.8)* |
| Depressive symptoms | 657 (11.5) | 0 (0.0) | 657 (18.6)* |
| Abnormal kidney function | 853 (17.7) | 0 (0.0) | 853 (26.5)* |
| Liver cirrhosis or steatosis | 2146 (54.6) | 0 (0.0) | 2146 (76.6)* |
| Cancer | 1080 (73.2) | 425 (71.8) | 655 (74.2) |
| Cancer within 3 prior months | 14 (0.9) | 0 (0.0) | 14 (1.6)* |
| Stroke within 12 prior months | 37 (0.7) | 9 (0.4) | 28 (0.8) |
| Stroke within 3 prior months | 5 (0.1) | 0 (0.0) | 5 (0.2) |
| Parkinson’s disease | 24 (0.4) | 5 (0.2) | 19 (0.6) |
| Cardiac disease within 3 prior months | 246 (4.3) | 0 (0.0) | 246 (6.9)* |
| Moderate to severe COPD | 419 (7.2) | 0 (0.0) | 419 (11.7)* |
| COPD within 3 prior months | 22 (1.6) | 0 (0.0) | 22 (2.3) * |
| Values are absolute numbers (%) or means (standard deviation). The high-risk group was based on recommendations from the RIVM and WHO, and included participants aged over 70 years, and participants with asthma and COPD, diabetes, cancer, participants with current use of antineoplastic and immunosuppressive agents, obesity (body mass index >30 kg/m^2^), end-stage kidney disease with estimated glomerular filtration rate <15 milliliters/minute, liver steatosis or cirrhosis, cardiac diseases (including myocardial infarction, heart failure, atrial fibrillation and revascularization).  Acute or unstable disease is defined as: Dementia diagnosis, diagnosis of moderate to severe Chronic obstructive pulmonary disease (COPD), current depressive symptoms, abnormal kidney function (defined as estimated Glomerular Filtration Rate <60 millilitre/minute), current liver disease (defined as liver steatosis, and liver cirrhosis). Diagnosis of the following within the previous 3 months: stroke, cancer (including antineoplastic agents), diabetes mellitus, COPD, cardiac disease (heart failure, myocardial infarct, atrial fibrillation, and revascularisation).  ^Genetic ancestry data was available in a random set of 87% of this study population. | | | |
| *P-value < 0.05 | | | |


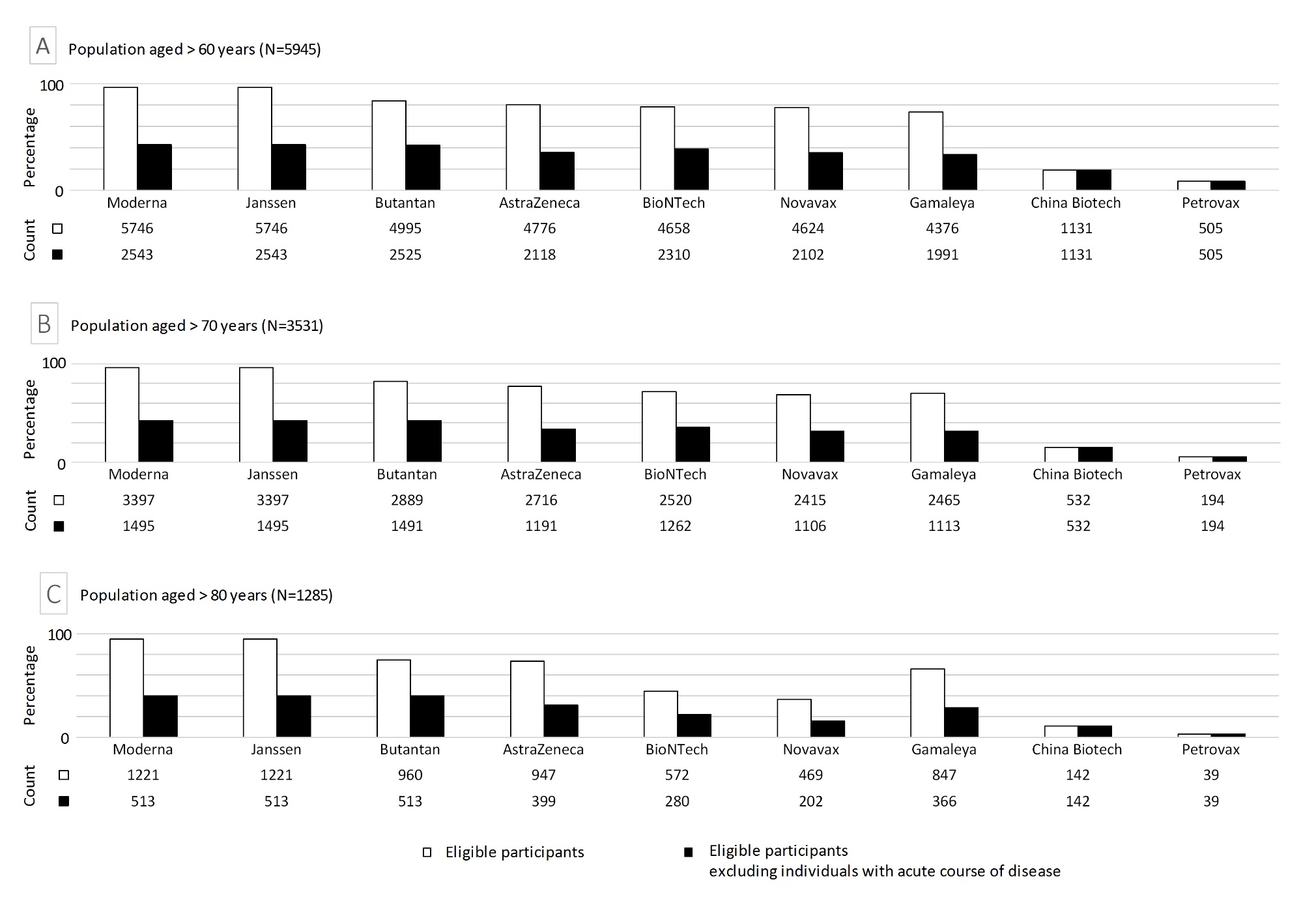


**Online Resource Figure 1 The number and proportion of eligibility from the Rotterdam Study population for ongoing clinical phase III trials on a vaccine against SARS-CoV-2.** White bars indicate data from the first analysis; black bars indicate data from the second analysis. The difference between these two analyses is the operationalization, and thus inclusion or exclusion, of the eligibility criteria ‘(un)stable disease’, ‘acute course of disease’, or ‘other condition increasing risk of participation’. A) Population aged > 60 years, B) population aged > 70 years and C) population aged > 80 years

**References**

1. RIVM. *Risicogroepen en COVID-19*. 2020 10-11-2020]; Available from: <https://www.rivm.nl/coronavirus-covid-19/risicogroepen>.

2. Ikram, M.A., et al., *The Rotterdam Study: 2018 update on objectives, design and main results.* Eur J Epidemiol, 2017. **32**(9): p. 807-850.

3. de Roos, E.W., et al., *Asthma and its comorbidities in middle-aged and older adults; the Rotterdam Study.* Respir Med, 2018. **139**: p. 6-12.

4. Terzikhan, N., et al., *Prevalence and incidence of COPD in smokers and non-smokers: the Rotterdam Study.* Eur J Epidemiol, 2016. **31**(8): p. 785-92.

5. Leening, M.J., et al., *Methods of data collection and definitions of cardiac outcomes in the Rotterdam Study.* Eur J Epidemiol, 2012. **27**(3): p. 173-85.

6. Vilagut, G., et al., *Screening for Depression in the General Population with the Center for Epidemiologic Studies Depression (CES-D): A Systematic Review with Meta-Analysis.* PLoS One, 2016. **11**(5): p. e0155431.

7. Ott, A., et al., *Prevalence of Alzheimer's disease and vascular dementia: association with education. The Rotterdam study.* BMJ, 1995. **310**(6985): p. 970-3.

8. Ligthart, S., et al., *Lifetime risk of developing impaired glucose metabolism and eventual progression from prediabetes to type 2 diabetes: a prospective cohort study.* Lancet Diabetes Endocrinol, 2016. **4**(1): p. 44-51.

9. Alferink, L.J.M., et al., *Coffee and herbal tea consumption is associated with lower liver stiffness in the general population: The Rotterdam study.* Journal of Hepatology, 2017. **67**(2): p. 339-348.

10. Darweesh, S.K.L., et al., *Trends in the Incidence of Parkinson Disease in the General Population.* American Journal of Epidemiology, 2016. **183**(11): p. 1018-1026.

11. Bots, M.L., et al., *Prevalence of stroke in the general population. The Rotterdam Study.* Stroke, 1996. **27**(9): p. 1499-501.

12. Ewing, J.A., *Detecting alcoholism. The CAGE questionnaire.* JAMA, 1984. **252**(14): p. 1905-7.

13. Abdin, E., et al., *A Non-Parametric Item Response Theory Evaluation of the CAGE Instrument Among Older Adults.* Subst Use Misuse, 2018. **53**(3): p. 391-399.
